# Clinical study of central cholinergic pathway damage in two mild cognitive impairment patients

**DOI:** 10.1007/s10072-021-05573-9

**Published:** 2021-09-16

**Authors:** Qing Liu, Ming Zhong, Shiqi Yuan, Chen Niu, Xiaoying Ma

**Affiliations:** 1 Department of Neurology, Guihang Guiyang Hospital, Guiyang, Guizhou, China; 2 Department of Medical Imaging, Guizhou Provincial People’s Hospital, Guiyang, Guizhou, China

**Keywords:** Mild vascular cognitive impairment, Amnestic mild cognitive impairment, The central cholinergic system, Cognitive dysfunction

## Abstract

**Objectives:**

To explore the role of the central cholinergic system in amnestic mild cognitive impairment (aMCI) and mild vascular cognitive impairment (vMCI).

**Methods:**

Twenty-five aMCI patients and 25 vMCI patients were enrolled in this study, and 25 healthy people were chosen as a control group. All participants performed a set of cognitive function scales and were subjected to a brain MRI. We analyzed differences in neuropsychological damage between groups, as well as the degree of brain atrophy and changes in the microstructure of central cholinergic pathways (CCP) in relation to effects on neuropsychological scores.

**Results:**

(1) Regarding neuropsychological characteristics of the three groups, scores on the MoCA scale, immediate memory, delayed recall, cued recall, long time prolonged recognition, and CDR-SB of the control group were significantly better than those of the aMCI and vMCI groups. Scores on immediate memory, delayed memory, cued recall, long time delayed recognition, and Forward of Digital Span Test (FDST) in the aMCI group were lower than those in the vMCI group. Compared with the aMCI group, the vMCI group was significantly delayed in Trail Making Test (TMA)-A, TMT-B, and TMT B-A. There were no significant differences in HAMA, HAMD, MMSE, MoCA, the Boston Naming Test (BNT), language fluency or visual scale of posterior atrophy (Koedam score) between the vMCI and aMCI groups. (2) As for microstructure changes in the central cholinergic pathway, vMCI group had a decreased FA value in the cingulum (Cing) of the medial pathway, but an increased MD value in the external capsule (Excap) of the lateral pathway when compared to other two groups. Furthermore, the CingMD value of the vMCI group was higher than that of the control group, but the difference was not obvious when compared to the aMCI group. (3) Last, we researched microstructural changes to CCP, degree of brain atrophy, and neuropsychological scores by using partial correlation analysis for all participants. CingFA was negatively correlated with TMT-B, B-A, and FDST. CingMD was negatively correlated with FDST. ExcapFA was positively correlated with MMSE and Backward of BDST, while ExcapMD was negatively correlated with MMSE and MoCA. Claustrum (Claus)FA was positively related to MoCA and FDST, but was negatively related to TMT-A. ClausMD was negatively correlated with MoCA and language fluency. Koedam score was positively correlated with CDR-SB, ExcapMD, and ClausMD, but negatively correlated with MMSE score and inverse BDST.

**Conclusion:**

The central cholinergic system is involved in the cognitive impairment of both aMCI and vMCI, and their mechanisms may be distinct. aMCI patients may present with primary CCP impairment while vMCI patients probably exhibit impairment secondary to vasogenic damage to the cholinergic system projection network. The lateral cholinergic pathway was more severely impaired than the medial pathway in vMCI patients, in addition to being associated with decreased executive and general cognitive functions. The damage to CCP was related to the degree of brain atrophy, and both may be involved in the development and progression of cognitive dysfunction.

## Introduction

Mild cognitive impairment (MCI) and dementia have high morbidity and common risk factors for Chinese patients [1]. The prevalence rate of MCI in those over 60 years old in China is estimated to be 15.5% (15.2–15.9%) and is predicted to reach 38.77 million. [1] Since the concept of MCI was proposed by Petersen [2] in the 1990s, a series of prospective studies have confirmed the predictive relationship between MCI and dementia. Although MCI has a high risk of transforming into dementia, not all cases of MCI proceed to dementia. [3] The incidence rate and clinical phenotype of MCI are affected by sociodemographic and clinical factors, such that the proportion of its prevalence may vary from country to country. Worldwide, about 5–15% of MCI cases eventually develop into dementia per year [4], but the ratio for Australian aboriginal residents of age ≥ 60 years old was 41%, [5] while about 44% [5]–50% [4] were stable MCI cases able to maintain their original cognitive levels at 6 years of follow-up. Even about 1 ~ 15% [4–6] of patients with stable MCI can be reverted to normal [4–6]. Notably, even if some MCI patients can maintain or even regain normal cognitive function, they are still at higher risk of developing dementia than those who have never been diagnosed with MCI [3]. Therefore, intervention at the MCI stage is the focus of dementia prevention and treatment.

The central cholinergic system may play a role in cognitive dysfunction. The innervation of cholinergic nerve in the whole brain is mediated through the medial and lateral pathways of central cholinergic pathways (CCP). The cholinergic input of the cerebral cortex comes from the nucleus basalis of Meynert (NbM) Ch4 region [7]. CCP project to global cortex following the medial (Cing) pathway through corpus callosum, and lateral pathway [8], which further divide into the capsule branches through the external capsule (Excap) and the lateral fissure branches through the claustrum (Claus). Some studies visually assessed white matter (WM) hyperintensity in cholinergic pathway by cholinergic pathway hyperintensity scale (CHIPS), suggesting WM lesions in the CCP caused by cerebral small vessel diseases may affect the volume changes of the cortex and subcortical structures in cholinergic distribution areas, [9, 10] thus influencing cognitive function. However, the mechanisms of how CCP is affected in amnestic mild cognitive impairment (aMCI) and mild vascular cognitive impairment (vMCI) remain unclear. One recent study has shown that the cholinergic system is affected differently based on the degree and location of local cortex atrophy [11]. Furthermore, the central cholinergic system can even resist the damage of hippocampus, thalamus, and entorhinal cortex by forming a network system, and reduce the damage of MCI caused by whole brain lesions, which may have a protective effect in MCI [12]. All above suggest that the role of the cholinergic system in cognitive disorders is heterogeneous. Therefore, clinical studies should be conducted to analyze the role of the central cholinergic system in cognitive impairment with respect to different types or stratifications of cognitive disorders.

Applications of diffusion tensor imaging (DTI) and probabilistic tractography in investigation of the WM microstructural change and tracking fiber connections in CCP have been proved to be effective by our previous research [13]. The purpose of this study was to investigate the role of central cholinergic system impairment in aMCI and vMCI by using DTI and probabilistic tractography analysis, as well as assessing the effects of brain atrophy on cognitive impairment in patients with two types of MCI.

## Methods

### Study population

A total of 25 patients aged 45 to 79 years old who were diagnosed with aMCI and vMCI were selected as research subjects between January 2018 and August 2020 from the Memory Clinic of Department of Neurology, Guihang Guiyang Hospital. A total of 25 age-matched healthy subjects who underwent physical examination at the physical examination center of Guihang Guiyang Hospital during the same period were assigned to the control group. All subjects were of Han nationality, with education nality, with educ completed a full range of cognitive function scale examinations and cranial magnetic resonance imaging (MRI) examinations. This study was performed in a double-blind controlled manner. Researchers involved in neuropsychological examinations, craniocerebral MRI scans, and imaging analysis were not apprised of the patients’ diagnosis. Age and education levels were selected as matching conditions between the AMCI, VMCI, and healthy control groups.

### aMCI group criteria

Individuals met the diagnostic criteria of MCI proposed by Petersen [2] and have characteristics as follows [14, 15]: (1) cognitive impairment with slow progression, mainly with episodic memory impairment, with/without functional decline in other cognitive domains; (2) medial temporal lobe atrophy (MTA) ≥ 1, Fazekas score ≤ 1; (3) cognitive dysfunction caused by other causes (such as cerebrovascular disease, infection, tumor, anxiety, and depression) were excluded.

### *vMCI group criteria *[13]

In accordance with the MCI diagnostic criteria proposed by Peterson [2], MRI examination: (1) multiple (≥ 3) supratentorial subcortical small infarcts (3–20 mm in diameter) with/without any degree of white matter lesions; moderate to severe white matter lesions (Fazekas score ≥ 2) [16], with/without small infarcts; subcortical infarcts in one or more critical sites such as putamen, globus pallidus, or thalamus; (2) no cortical or watershed infarct, cerebral hemorrhage, obstructive, normal cranial pressure hydrocephalus, or specific white matter lesions (such as multiple sclerosis, sarcoidosis, or radio-encephalopathy); (3) no hippocampus and entorhinal cortex atrophy with MTA = 0 and Hachinski > 4 points.

### Control group criteria

No history of neurological or psychiatric diseases, no systemic diseases associated with cognitive impairment (such as liver and kidney insufficiency, hypothyroidism, vitamin deficiency), no history of alcohol or drug abuse, no cognitive impairment, MMSE score > 27 points, Montreal Cognitive Assessment Scale (MoCA) score > 25, no dementia, CDR = 0, no abnormalities in head MRI, Fazekas = 0, MTA = 0.

### Exclusion criteria

(1) Severe visual and hearing impairment, severe aphasia, disability, or other reasons for failure to complete the neuropsychological assessment; (2) other diseases that may affect cognitive function (such as encephalitis, epilepsy, tumor, hypothyroidism; (3) years of education < 6 years, Hamilton Anxiety Scale (HAMD) > 17 points, Hamilton Depression Scale (HAMA) > 11 points, schizophrenia or severe cognitive dysfunction (unable to complete the neuropsychological test); (4) recent stroke within 3 months; (5) inflammatory microvascular disease; (6) clinically confirmed diseases of the gastrointestinal tract, kidney, liver, or respiratory system; infectious diseases, endocrine system diseases, cardiovascular system diseases (atrial fibrillation, history of myocardial infarction, cardiac dysfunction), cancer, alcoholism, drug addiction, use of drugs that may affect cognitive function: including sedatives and hypnotics, anti-anxiety drugs, intelligence agents, cholinoid preparations, celery allergy; (7) severe claustrophobia; (8) those unable to undergo MRI examination.

This study was approved by the Ethics Committee of Guihang Guiyang Hospital (No.: GK2017050001), and all patients or their proxies provided informed consent.

## Clinical data collection

### Baseline characteristics

Sex, age, education level, and previous medical history (cerebrovascular disease, hypertension, hyperlipidemia, diabetes) of all subjects were recorded.

### Neuropsychological assessment

Montreal Cognitive Assessment (MoCA), the MMSE, Auditory Verbal Learning Test (AVLT), Boston Naming Test (BNT), the Trail Making Test A and B, Forward of Digital Span Test (FDST), Backward of Digital Span Test (BDST), Hachinski Ischemia Scale (HIS), Activities of Daily Living (ADL), Clinical Dementia Rating scale (CDR), HAMA, HAMD, MTA scale, Koedam score, and Fazekas scale were completed. Cognitive function tests were assessed by one neuropsychological examiner who was unaware of the patients’ diagnosis (patients with a history of stroke were assessed at least six months after stroke).

### Imaging evaluation

All patients received a 1.5Tesla (1.5 T Siemens Trio TIM) head MRI scan, including T1W, T2W, FLAIR T2W, and DTI sequence imaging. We excluded patients with brain lesions other than cerebral atrophy. All MRI examinations were performed within 1 week after the neuropsychological evaluation, and there were no new stroke events or TIAs during evaluation.

#### Ordinary scanning

1.5 T GE HDE superconducting MRI scanning system was used for image acquisition, and 8-coil standard head coils were used as transmitting and receiving coils. Imaging data acquisition includes the following parts: T1 structural image, DTI, and FLAIR T2-weighted image. T1 imaging of brain structure was performed by 2D-SE/IR sequence scanning. Scanning parameters were as follows: T1 flair repetition time (TR) 2221.44 ms, echo time (TE) 12.15 ms, reverse rotation angle 90°, Ti = 750 ms, layer thickness 6 mm, and isotropic voxels covering the whole brain scan. DTI sequence parameters were as follows: TR 9000 ms, TE 129.30 ms, 90°, FOV 240 mm^2^, matrix = 256 × 256, layer thickness 5 mm, continuous axial scanning 40 layers, which were obtained from 16 nonlinear coding directions (B = 1000 s/mm^2^) and one non-dispersive weighted image (B = 0 s/mm^2^, B0). Fazekas score was obtained from FLAIR image, TR 8002 ms, TE 123.9 ms, and reversal time 2000 ms, FOV 240 mm^2^, layer thickness of 5 mm, layer spacing of 1 mm, layer number of 16 layers. DTI images were collected by extending the linear AC-PC line, and some cerebellar images (including the cerebellar cortex) of the subjects were omitted.

#### DTI imaging analysis

DTI data analysis: (1) FSL software was used to perform “eddy current correction,” “artifact removal,” and “non-brain tissue removal,” and B0 images of all DTI data were extracted respectively. B0 image on DTI sequence of one subject was selected, and the SYN registration method of ANTS was used for registering the B0 image of the subjects to the EPI template of MNI standard space (SPM8 template); the registration results were good. The B0 images of all subjects were registered to the generated and registered B0 images of the first subject by ANTS. Due to the clear structure of the registered B0 image, the later image registration results were also relatively good. After that, all registered B0 images were averaged to generate B0 templates. (2) DTI image registration: 4D DTI images were converted into multiple 3D DTI images by selecting a DTI imaging with better image quality in the direction and registering it on the B0 template. ANTS was used to register images of each subject in 16 directions to the standard B0 template. All registered 3D images were combined into 4D DTI images. (3) DSI Studio (http://dsi-studio.labsolver.org) fiber tracking: a total of 15 diffusion sampling directions were obtained by DTI diffusion scheme. The *b*-value was 1000 s/mm^2^. The NbM ROIs were according to a previous research [17]. The bilateral cingulum (Cing) and bilateral external capsule (ExCap) ROIs were selected automatically based on Human Connectome Project dMRI population-averaged template (HCP-1065 atlas) loaded into the DSI studio program (Graph 3). The bilateral claustrum (Claus) ROIs were defined in each participant’s native space. The in-plane resolution was 0.9375 mm. The slice thickness was 5 mm. The tracking threshold was 0.3. A deterministic fiber tracking algorithm was used. The angular threshold was 90°. The step size was 0.98 mm and a total of 200,000 seeds were placed. Fiber trajectories were smoothed by averaging the propagation direction with 30% of the previous directions. Tract was set between 30 and 300 mm. The FA and MD values of the seed site fiber were then calculated through software.

#### ***Koedam’s score ***[18]

The overall score based on the presence of atrophy in sagittal, axial, and coronal orientation was assessed for left and right sides separately on sagittal and coronal sections of the T1-weighted sequence and axial sections of the FLAIR sequence. The following anatomical landmarks were rated in three different orientations: (a) sagittal orientation: widening of the posterior cingulate and parieto-occipital sulcus, and atrophy of the precuneus on left and right by considering paramedian sagittal images; (b) axial orientation: widening of the posterior cingulate sulcus and sulcal dilatation in parietal lobes on axial images; (c) coronal orientation: widening of the posterior cingulate sulcus and parietal lobes on coronal images. In case of different scores on different orientations, the highest score was considered. Grade 0: no cortical atrophy-closed sulci of parietal lobes and cuneus; Grade 1: mild cortical atrophy-mild widening of posterior cingulate and parieto-occipital sulcus; Grade 2: substantial cortical atrophy-substantial widening of the sulci; Grade 3: end-stage “knife blade” trophy-extreme widening of the sulci.

### Statistical methods

SPSS 23.0 statistical software package was used for data analysis. Demographics were compared between groups using independent *t-*test for age and education and chi-square test (*χ*^2^ test) for gender. As the CDR rating was 0.5 for each MCI participant and 0 for control participants, we used the CDR sum of boxes (CDR-SB) for group comparisons in a one-way analysis of variance with covariates (ANCOVA), controlling for influencing factors such as age, gender, education, hypertension, and diabetes. A similar procedure was conducted for each neuropsychological test and the group difference in diffusivity indices. *P* < 0.05 was considered statistically significant.

We investigated the contribution of the damage in the cholinergic system to cognitive impairment in aMCI and vMCI by using probabilistic tractography analysis. We tracked the fibers along with the two major CCP. To test whether CCP contributed to neuropsychological scores across groups, we performed a partial correlation between neuropsychological scores, FA and MD in CCP, controlling for age, gender, education, hypertension, and diabetes.

## Results

### Baseline data comparison

Comparisons between the three groups are shown in Table 1. There were no significant differences in age, education, sex ratio, ADL score, MMSE, Boston naming test, language fluency, or number breadth among the three groups. The number of patients with hypertension history in the vMCI group was significantly higher than for the aMCI and control groups, and this difference was statistically significant. Although there were no statistically significant differences in Koedam scores among the three groups, Hachinski and Koedam scores for the vMCI group were significantly higher than those for the control group, suggesting the presence of remarkable vascular risk factors and more serious posterior cerebral cortex atrophy in the vMCI group. FA values for the cingulate branch of the medial pathway and MD values for the lateral fissure branch of the lateral pathway also showed statistically significant differences between the three groups.

**Table 1:** Comparisons between groups

	Control	aMCI	vMCI	*t*/***χ***^2^/*F*
Sample size	27	25	25	
Age*years	60 .8 (8.7)	62.3 (10.5)	65.6 (8.3)	0.90
Education (years)	11.40 (4.2)	9.40 (2.7)	11.60 (3.1)	1.80
Male/female (%)	29.4/70.6	46.7/53.3	54.5/45.5	2.0
Hypertension (%)	29.4	20.0	81.8	11.4**
Diabetes (%)	0	6.7	27.3	6.10*
HIS	0.29 (0.47)	0.07 (0.26)	3.27 (2.65)	20.99***
Koedam score	0.76 (0.56)	1.20 (0.86)	1.45 (0.93)	2.85
MMSE	28.59 (1.84)	26.80 (2.68)	27.18 (3.28)	2.15
MoCA	26.24 (3.01)	22.47 (4.98)	22.64 (3.83)	4.40*
ADL	20.0 (0)	20.1 (0.3)	20.5 (1.0)	2.6
TMT-A	52.94 (22.36)	66.27 (19.18)	98.27 (28.01)	13.26***
TMT-B	77.35 (27.51)	111.33 (32.59)	150.09 (43.27)	15.59**
TMT B-A	24.76 (24.32)	61.93 (43.72)	52.09 (29.78)	5.23**
BNT	28.65 (10.04)	22.80 (5.97)	21.82 (7.57)	3.05
FDST	9.12 (0.99)	8.33 (1.35)	8.55 (1.23)	1.95
BDST	5.06 (1.75)	4.00 (1.13)	5.45 (1.70)	3.23*
Language influence	16.18 (3.34)	13.87 (4.19)	15.45 (3.48)	1.60
AVLT_Immediate memory	24.35 (4.92)	11.73 (3.86)	19.00 (4.45)	31.99***
AVLT_Delayed recall	8.47 (2.00)	5.07 (2.79)	7.82 (3.19)	7.25**
AVLT_Cue	10.41 (2.24)	6.93 (2.28)	7.45 (2.62)	9.99***
AVLT_Delayed recognition	13.06 (1.64)	10.80 (3.41)	10.55 (2.46)	4.36*
CDR-SB	0.03 (0.12)	0.93 (0.53)	1.64 (1.14)	20.88***
Cing-FA	0.57 (0.05)	0.54 (0.05)	0.49 (0.06)	7.99***
Excap-FA	0.38 (0.02)	0.36 (0.03)	0.36 (0.03)	1.39
Claus-FA	0.35 (0.04)	0.32 (0.05)	0.32 (0.05)	2.27
Cing-MD	0.99 (0.10)	1.10 (0.22)	1.14 (0.15)	3.17
Excap-MD	1.07 (0.08)	1.14 (0.15)	1.25 (0.12)	6.82**
Claus-MD	1.16 (0.10)	1.21 (0.19)	1.28 (0.16)	2.00

^*^P ≤ .005, **P < 0.01, ***P < 0.001

### Comparisons between groups

The total score for each CDR domain was used as a semi-quantitative indicator; the control group value was 0, and the value in the MCI group was 0.5. Since changes in CDR-SB were easier to observe and compare, CDR-SB was selected for subsequent statistical analysis.

#### Neuropsychological comparisons

MOCA scale score, immediate memory, delayed recall, cued recall, long time prolonged recognition, and CDR-SB for control group were significantly better than for the aMCI and vMCI groups. Scores for immediate memory, delayed memory, cued cue, long time delayed recognition, and BDST for the aMCI group were lower than for the vMCI group. Compared with the aMCI group, the vMCI group was significantly delayed in TMT-A, B, and B-A. However, there were no significant differences in HAMA, HAMD, MMSE, MOCA, and BNT, language fluency or visual scale of posterior atrophy (Koedam) between the vMCI and aMCI groups.

#### ***Comparison of DTI parameters for the CCP (Table ******2******, Graphs******1******and******2******)***

The vMCI group had a decreased CingFA value for the medial pathway, but an increased ExcapMD value for the lateral pathway when compared to other two groups. Furthermore, the CingMD value for the vMCI group was higher than for the control group, but the difference was not obvious when compared to the aMCI group. These results indicate that patients in the vMCI group show more severe damage to both internal and lateral cholinergic pathways than those in the aMCI and control groups.

**Table 2:** Relationships between FA, Koedam, and neuropsychological scores

Neuropsychological Scores	CingFA	ExcapFA	ClausFA	Koedam score
CDR-SB	− 0.25	− 0.60	− 0.26	0.34*
MoCA	0.002	0.17	0.36*	− 0.30
MMSE	0.02	0.33*	0.38*	− 0.34*
AVLT-Immediate memory	0.16	0	− 0.05	− 0.16
AVLT-Delayed recall	0.04	0.19	0.06	− 0.17
AVLT-Cue	0.07	0.11	0.27	− 0.23
AVLT-Delayed recognition	0.16	0.31	0.12	− 0.70
TMT-A	− 0.31	− 0.06	− 0.41*	0.25
TMT-B	− 0.45**	− 0.15	− 0.15	0.11
TMT B-A	− 0.42*	− 0.16	0.13	− 0.24
BNT	0.30	0.12	− 0.14	0.04
FDST	0.06	0.41*	0.30*	− 0.52
BDST	0.02	0.17	0.15	− 0.36*
Language influence	− 0.08	0.09	0.23	− 0.29
Koedam score	− 0.06	− 0.12	− 0.44**	/

^*^P ≤ *.05, **P < 0.01, ***P < 0.001

**Graph 1:**
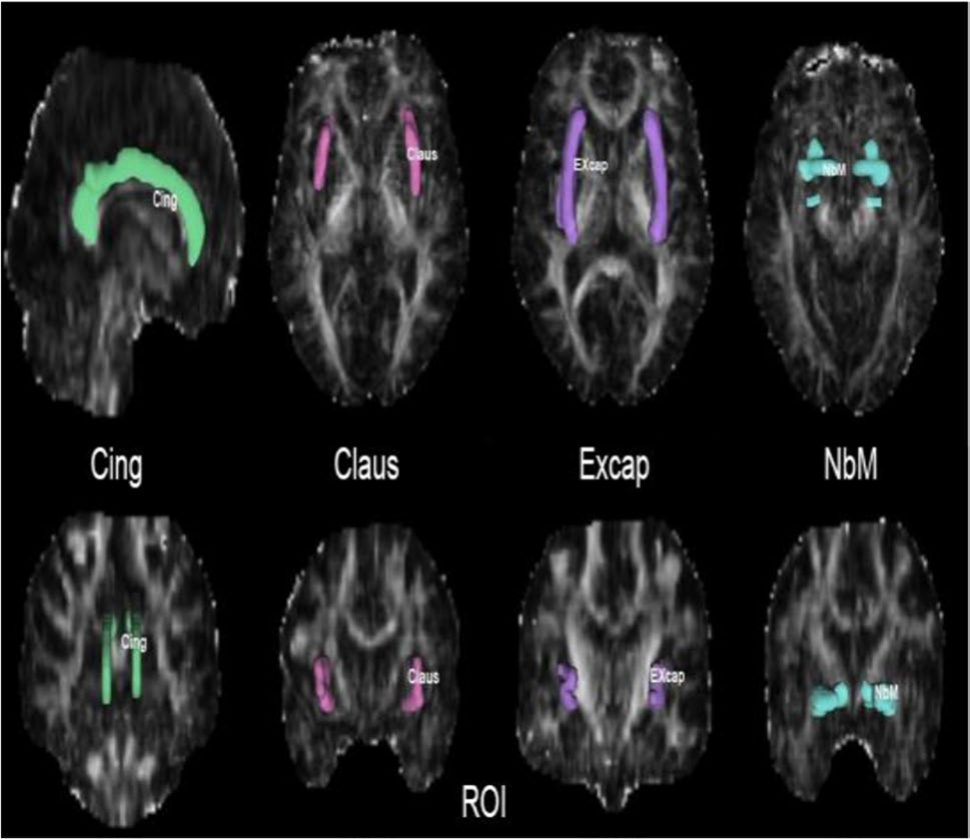
3D imaging of cholinergic pathway. Light blue-seed ROI of NbM; green-Medial pathway(Cing); pink-Claus branch of lateral pathway; purple-Excap branch of lateral pathway

**Graph 2:**
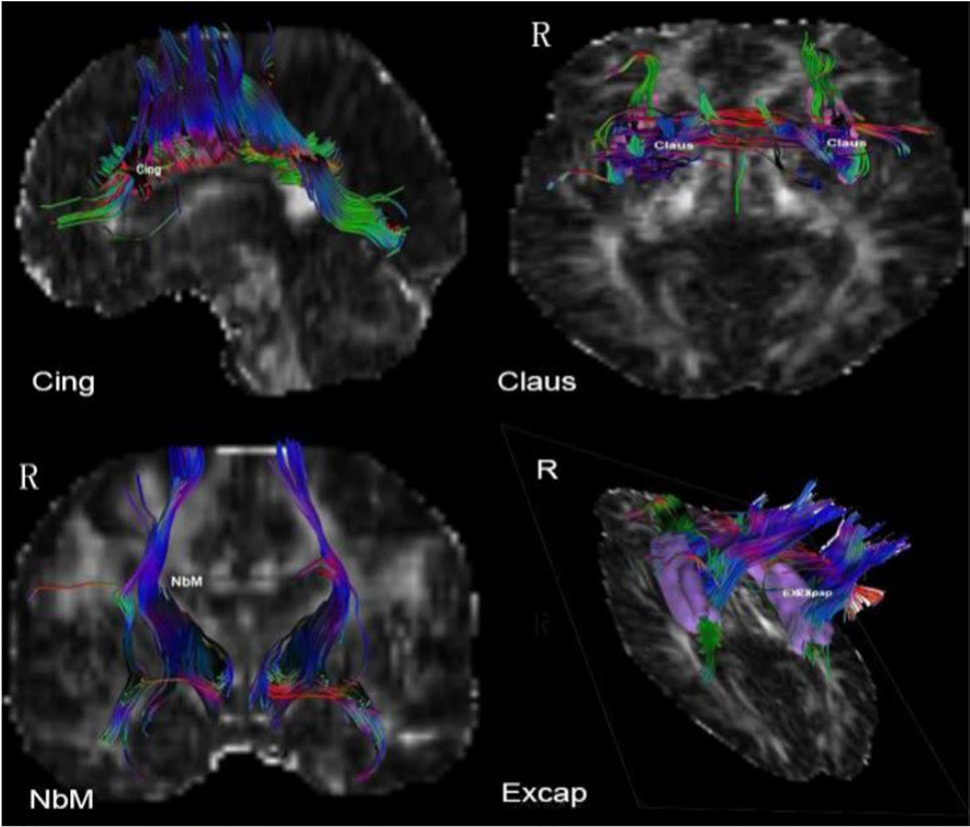
Tracing imaging of cholinergic pathway. Light blue-seed ROI of NbM; green-Medial pathway(Cing); pink-Claus branch of lateral pathway; purple-Excap branch of lateral pathway

### *Relationships between microstructural CCP changes, brain atrophy degree, and neuropsychological scores (Table **3**)*

**Table 3:** Relationships between MD, Koedam, and neuropsychological scores

Neuropsychological scores	CingMD	ExcapMD	ClausMD
CDR-SB	− 0.09	0.23	0.30
MoCA	− 0.27	− 0.26	− 0.42*
MMSE	− 0.24	− 0.19	− 0.29
AVLT-Immediate memory	− 0.10	− 0.12	− 0.07
AVLT-Delayed recall	− 0.32	− 0.18	− 0.01
AVLT-Cue	− 0.23	− 0.16	− 0.18
AVLT-Delayed recognition	− 0.07	− 0.28	− 0.28
TMT-A	0.12	− 0.14	0.04
TMT-B	0.09	0.07	0.19
TMT B-A	0.20	0.19	0.21
BNT	− 0.16	− 0.13	− 0.19
FDST	− 0.44*	− 0.03	0.01
BDST	− 0.17	0.35*	0.17
Language influence	− 0.14	− 0.01	− 0.31
Koedam score	− 0.12	0.14	0.31

^*^P ≤ .31a, **P < 0.01, ***P < 0.001

*HIS*, Hachinski Ischemia Scale; *MMSE*, Mini-mental State Examination; *MoCA*, Montreal Cognitive Assessment; *ADL*, Activities of Daily Living; *TMT-A*, Trail Making Test A; *TMT-B*, Trail Making Test B; *BNT*, Boston Naming Test; *FDST*, Forward of Digital Span Test; *BDST*, Backward of Digital Span Test; *AVLT*, Auditory Verbal Learning Test; *CDR-SB*, Clinical Dementia Rating scale Sum of Boxes; *Cing-FA*, the FA values of medial pathway through corpus callosum; *Excap-FA*, the FA values of lateral pathway through external capsule; *Claus-FA*, the FA values of lateral fissure branches through the claustrum. *Cing-MD*, the MD values of medial pathway through corpus callosum; *Excap-MD*, the MD values of lateral pathway through external capsule; *Claus-MD*, the MD values of lateral fissure branches through the claustrum

**Graph 3:**
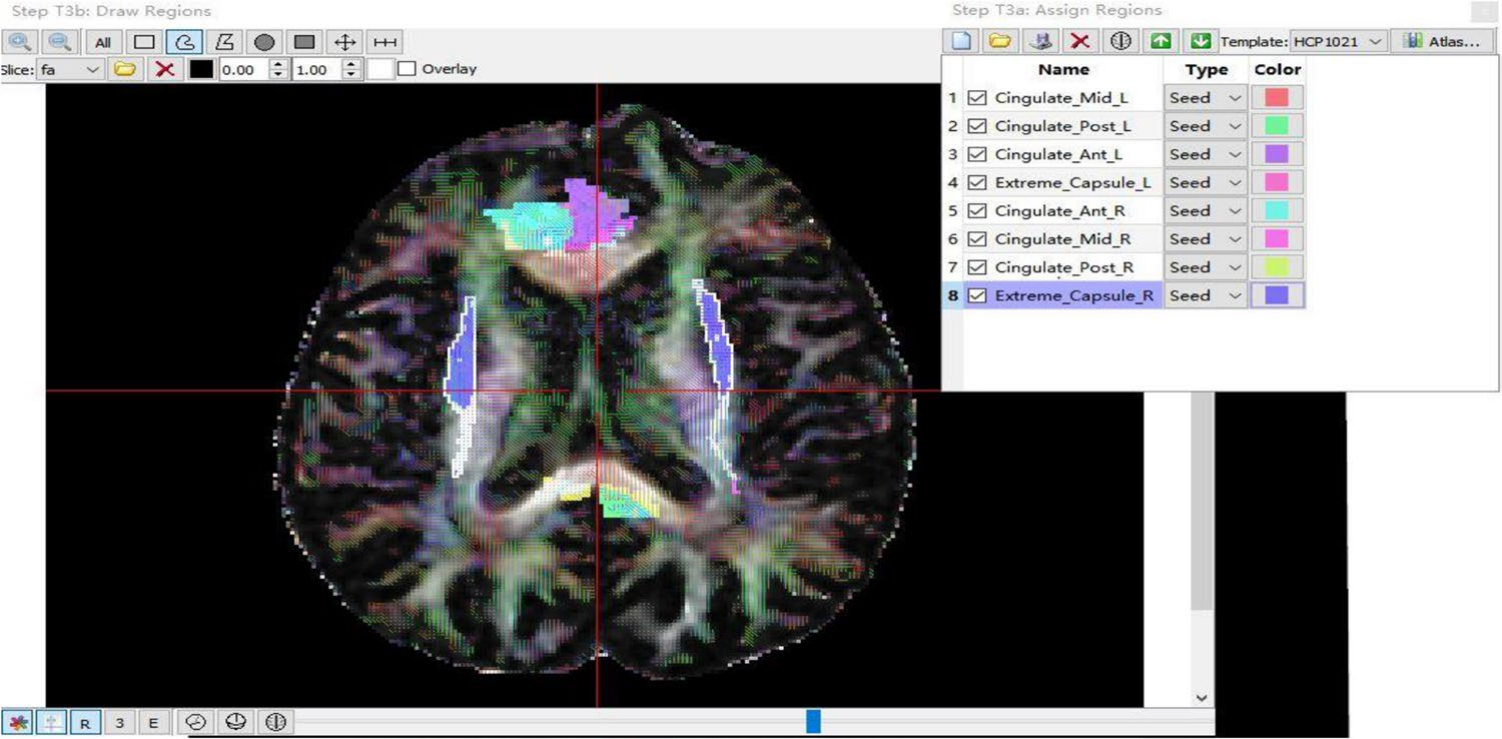
Cing and ExCap atlas continuous loaded into the DSI studio software

**Fig. 1:**
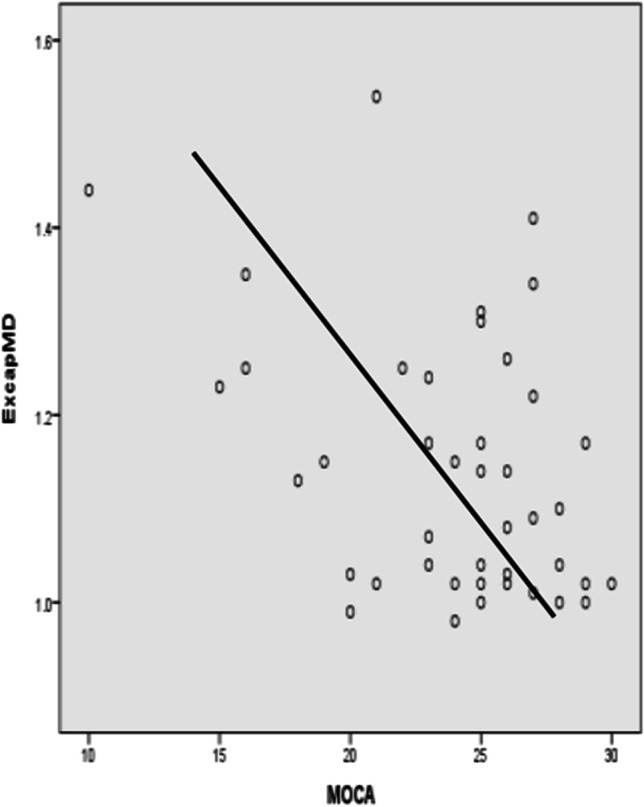
Scatter plot of ExcapMD and MoCA

**Fig. 2:**
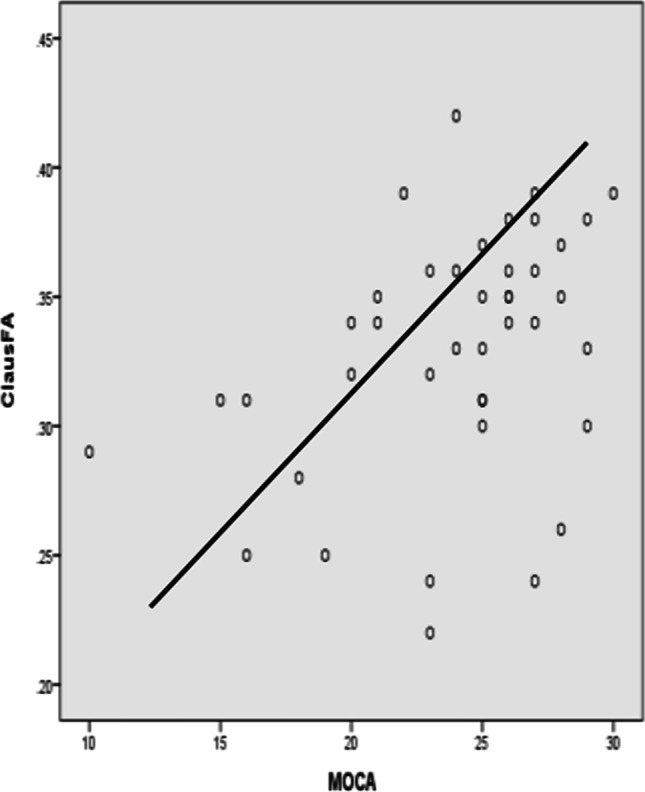
Scatter plot of ClausFA and MoCA

**Fig. 3:**
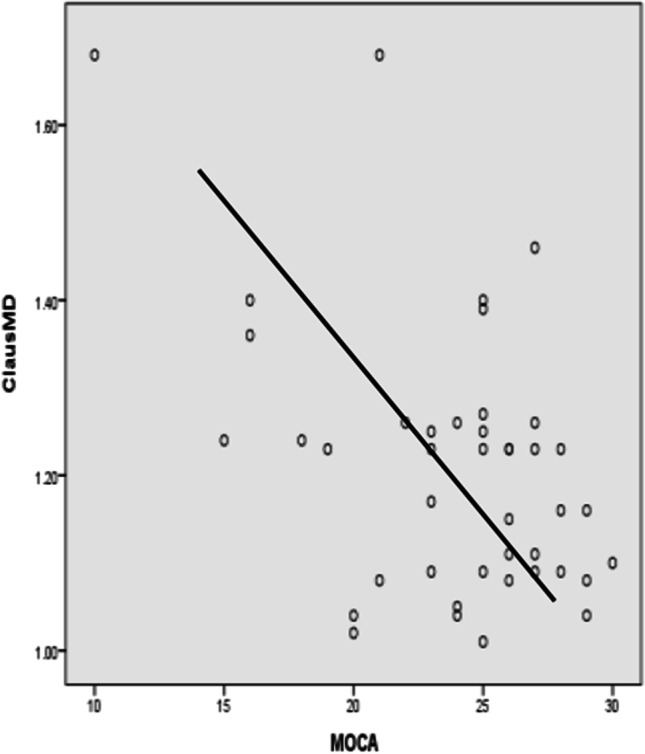
Scatter plot of ClausMD and MoCA

After adjusting for gender, age, years of education, hypertension, diabetes, and other factors (Graph 3), CingFA was negatively correlated with TMT-B and B-A. CingMD was negatively correlated with FDST. ExcapFA was positively correlated with MMSE and BNST, while ExcapMD was negatively correlated with MMSE and MoCA. ClausFA was positively related to MoCA and FDST, while negatively related to TMT-A. ClausMD was negatively correlated with MoCA and language fluency. Koedam score was positively correlated with CDR-SB, ExcapMD, and ClausMD, but negatively correlated with MMSE score and BNST (Figs. 1, 2 and 3).

## Discussion

Alzheimer’s disease (AD) and vascular cognitive impairment (VCI) are two of the most common cognitive disorders seriously affecting human life and health. For these cases, the focus of intervention is during pre-dementia, currently identified at the MCI stage. The etiology of MCI includes neurodegenerative diseases, cerebrovascular diseases, systemic diseases, poisoning, and trauma, [19]. In 2011, NIA-AA proposed the concept of AD-derived MCI based on etiological classification, indicating that patients at this stage of MCI would eventually develop to the end stage of AD, and suggested, for the first time, Aβ, Tau protein, and biomarkers indicative of neuronal damage as diagnostic criteria for AD-derived MCI [20]. This proposal lays a foundation for biomarkers to become reliable evidence in the clinical diagnosis of dementia. Biomarkers have become reliable evidence for the clinical diagnosis of dementia, but it remains difficult to obtain biological markers in clinical practice. Therefore, in clinical practice, structural MRI, functional MRI, PET, SPECT, and other imaging techniques are often used to screen out patients with pre-dementia or to predict the outcome of MCI by monitoring changes in brain morphology and brain function before patients show obvious symptoms of cognitive impairment. The medial temporal lobe (hippocampus and medial olfactory cortex) is the first area of atrophy observed in patients with AD, and the degree of atrophy may indicate disease progression. Studies have shown that the pathological manifestations of MCI patients with a high risk of transforming into AD are similar to those of patients with AD, with both showing a significant reduction in the volume of the medial temporal lobe and the parietal lobe upon structural MRI. Therefore, some scholars have proposed that the observation of temporal and parietal lobe atrophy on structural MRI could be a reliable predictor of the transition from MCI to AD [18]. AMCI patients with single-domain or multidomain lesions are more likely to progress to AD if an underlying degenerative cause is present [21]. Therefore, we selected MCI patients with multi-cognitive domain impairment, primarily due to episodic memory impairment with Fazekas score ≤ 1, and excluded cognitive dysfunction caused by cerebrovascular disease and other reasons, combined with medial temporal lobe atrophy (MTA ≥ 1) shown on structural MRI; this group was designated as the aMCI group for this study. From a clinical point of view, this group most closely resembles the clinical characteristics of MCI caused by AD (AD-derived MCI). In our study, almost all neuropsychological assessments of the control group showed significantly better performance than those of the aMCI and vMCI groups. We applied AVLT to test the memory function in aMCI and found that scores for immediate memory, delayed memory, cued cue, long time delayed recognition, and BDST of the aMCI group were lower than those of the vMCI group. These memory tests are highly sensitive for the diagnosis of preclinical AD according to clinical study [22]; it also showed our enrolled patients were closed to AD-derived MCI. Compared with the aMCI group, the vMCI group was significant delayed in TMT-A, B, and B-A. Our study showed that both aMCI patients and vMCI patients presented with multi-cognitive impairment, but there were significant differences in early cognitive impairment between the two kinds of MCI patients. The aMCI patients were marked by memory decline, while vMCI patients were mainly characterized by executive dysfunction, including disruption of attention, anti-interference ability, and information processing speed, at early stages. Meanwhile, memory function was relatively retained in vMCI patients, as the cognitive impairment of vMCI patients is partly caused by the loss of cortical neurons due to vascular disease or damage to prefrontal-subcortical circuits and thalamic-cortical circuits, or as a result of the disruption of extensive cortical-subcortical structural connections. [23] Taler [24] evaluated the functions of word acquisition, retrieval, and semantic information recognition in MCI patients and in elderly individuals with normal cognition, and found that the control group was superior to the MCI group in all tests, especially in the task of picture naming. Therefore, Taler believed that the picture naming test was the most likely language test item to reveal language defects in MCI patients. Therefore, in our study, the Boston naming test was used to evaluate language function in aMCI, vMCI, and control groups. This assessment showed no significant differences in language function between the three groups, suggesting that the impairment of language function in patients with aMCI and vMCI should be expected to follow impairments in memory, attention, information processing speed, and executive functions.

The central cholinergic nervous system is an important structure underlying learning, memory, and other cognitive processes. The dysfunction of acetylcholine in central neurons is an essential factor that promotes the decline of cognitive function in AD and aging [25]. Clinical studies have confirmed that damage to the central cholinergic pathway plays an important role in patients with vMCI. [13] We observed that, compared with the control group and the aMCI group, the vMCI group suffered more serious damage in all central cholinergic projection pathways. This effect was particularly distinct in the lateral pathway, which passes through positions prone to cerebrovascular disease, suggesting the central cholinergic pathways are more likely to be damaged by vascular disease. This mechanism may be fundamentally different from that observed in AD, for which cholinergic insufficiency is caused by primary cholinergic nucleus damage. Further analysis showed that the vMCI group was characterized by damage to internal and external central cholinergic projection pathways apparently associated with overall cognition (MMSE, MoCA), executive function (TMT-B, B-A and FDST, BDST), language fluency, and degree of brain atrophy, and somewhat affected by age, gender, level of education, hypertension, and diabetes. This finding is consistent with the views of Nemy et al. [26] Our study further supports the idea that the integrity of the central cholinergic projection system plays an important role in both normal aging and in cognitive decline associated with neurological diseases (including AD and VCI), while the mechanisms may be different. Patients with AD may have primary central cholinergic nucleus impairment. However, central cholinergic dysfunction in VCI patients is secondary to vasogenic damage of the cholinergic system and disconnection of the cortical and subcortical cholinergic network system [27].

Although our study found no significant differences in brain atrophy scores between groups, pairwise comparisons between each group showed that the Hachinski and Koedam scores of the vMCI group were significantly higher than those of the aMCI group and the control group. These results indicate that the vMCI group had significant vascular risk factors and cortical atrophy tendency. Koedam scores were correlated with overall cognitive level (CDR-SB, MMSE), executive function (BDST), and degree of damage to the lateral cholinergic pathway (ExcapMD, ClausMD). Therefore, the more severe the damage to the cholinergic pathway, the more severe the degree of brain atrophy, and the more obvious the decline in cognitive function. Wang et al. [28] explored the relationship between cholinergic pathway dysfunction and cerebral cortical structural changes in patients with different degrees of cognitive impairment accompanied by white matter lesions, finding that the thickness and volume of the left hemisphere cortex decreased significantly in patients with white matter lesions and cognitive dysfunction, consistent with the degree of cholinergic pathway damage. Taken in concert with our results, we suggest that this impairment of the cholinergic pathway is associated with decreased cerebral cortical structure and impairment of both overall cognitive function and executive function. Moreover, damage to the cholinergic pathway is related to the degree of brain atrophy, and we speculate that both factors may be involved in the occurrence and progression of cognitive dysfunction.

## Deficiency

There are limitations to our study. First, the small sample size of the study may bias the research results. Further studies with large sample sizes will help clarify the uncertainty of the results. Second, the vast majority of cholinergic axons emitted by NBM are unmyelinated fibers, and DTI technology cannot distinguish between myelinated and unmyelinated fibers. Therefore, DTI in combination with magnetic resonance spectroscopy imaging methods to study the neurochemical changes of cholinergic fibers would be helpful to better reveal pathologic changes to cholinergic pathways. Third, autopsy pathological studies have shown [29] that VCI and other neurodegenerative diseases often exist in the elderly, and there may be considerable overlap between VaD and AD. Therefore, we cannot rule out the possibility that patients enrolled in this study may include those with other preclinical cognitive dysfunction diseases. Diagnostic biomarkers should be added to the inclusion conditions of future experimental design to ensure the “simplicity” of the research objects. Finally, this study is a cross-sectional design, so it is not possible to make causal inferences based on this study. Rather, it needs to be further evaluated by longitudinal design and follow-up studies to assess the impact of changes to the cholinergic system on MCI cognitive function. In conclusion, subsequent neuroimaging studies on the effects of the cholinergic nervous system on MCI cognitive function and corresponding neuropsychiatric disorders will be of great significance.

## Data Availability

The datasets used and/or analyzed in the current study are available from the corresponding author on reasonable request.

## References

[1] Jia L, Du Y, Chu L (2020). Prevalence, risk factors, and management of dementia and mild cognitive impairment in adults aged 60 years or older in China: a cross-sectional study[J]. Lancet Public Health.

[2] Petersen RC, Smith GE, Waring SC (1997). Aging, memory, and mild cognitive impairment[J]. Int Psychogeriatr.

[3] Petersen RC, Lopez O, Armstrong MJ (2018). Practice guideline update summary: Mild cognitive impairment: report of the Guideline Development, Dissemination, and Implementation Subcommittee of the American Academy of Neurology[J]. Neurology.

[4] Dunne RA, Aarsland D, O’Brien JT (2021). Mild cognitive impairment: the Manchester consensus[J]. Age Ageing.

[5] Derrig H, Lavrencic LM, Broe GA (2020). Mild cognitive impairment in Aboriginal Australians[J]. Alzheimers Dement (N Y).

[6] Gomersall T, Smith SK, Blewett C (2017). ‘It’s definitely not Alzheimer’s: perceived benefits and drawbacks of a mild cognitive impairment diagnosis[J]. Br J Health Psychol.

[7] Mesulam MM, Geula C, Moran MA (1987). Anatomy of cholinesterase inhibition in Alzheimer’s disease: effect of physostigmine and tetrahydroaminoacridine on plaques and tangles[J]. Ann Neurol.

[8] Selden C, Roberts E, Stamp G (1998). Comparison of three solid phase supports for promoting three-dimensional growth and function of human liver cell lines[J]. Artif Organs.

[9] Lim JS, Kwon HM, Lee YS (2019). Effect of cholinergic pathway disruption on cortical and subcortical volumes in subcortical vascular cognitive impairment[J]. Eur J Neurol.

[10] Liu Y, Wu L, Yang C (2020). The white matter hyperintensities within the cholinergic pathways and cognitive performance in patients with Parkinson’s disease after bilateral STN DBS[J]. J Neurol Sci.

[11] Machado A, Ferreira D, Grothe M J, et al. The cholinergic system in subtypes of Alzheimer’s disease: an in vivo longitudinal MRI study[J]. Alzheimer’s Research & Therapy. 2020, 12(1). 10.1186/s13195-020-00620-7 PMC7203806 32375872

[12] Berlot R, Koritnik B, Pirtosek Z (2021). Preserved cholinergic forebrain integrity reduces structural connectome vulnerability in mild cognitive impairment[J]. J Neurol Sci.

[13] Liu Q, Zhu Z, Teipel S J, et al. White matter damage in the cholinergic system contributes to cognitive impairment in subcortical vascular cognitive impairment, no dementia[J]. Frontiers in Aging Neuroscience. 2017, 9. 10.3389/fnagi.2017.00047 PMC5326769 28289381

[14] Jack CR, Bennett DA, Blennow K (2018). NIA-AA Research Framework: toward a biological definition of Alzheimer’s disease[J]. Alzheimer’s & Dementia.

[15] Sperling RA, Aisen PS, Beckett LA (2011). Toward defining the preclinical stages of Alzheimer’s disease: recommendations from the National Institute on Aging-Alzheimer’s Association workgroups on diagnostic guidelines for Alzheimer’s disease[J]. Alzheimer’s & Dementia.

[16] Fazekas F, Chawluk JB, Alavi A (1987). MR signal abnormalities at 1.5 T in Alzheimer’s dementia and normal aging[J]. AJR Am J Roentgenol.

[17] Kilimann I, Grothe M, Heinsen H (2014). Subregional basal forebrain atrophy in Alzheimer’s disease: a multicenter study[J]. J Alzheimers Dis.

[18] Kaushik, Vani K, Chumber S (2021). Evaluation of MR visual rating scales in major forms of dementia[J]. J Neurosci Rural Pract..

[19] Anderson ND (2020). State of the Science on mild cognitive impairment[J]. J Gerontol B Psychol Sci Soc Sci.

[20] Mckhann GM, Knopman DS, Chertkow H (2011). The diagnosis of dementia due to Alzheimer’s disease: recommendations from the National Institute on Aging-Alzheimer’s Association workgroups on diagnostic guidelines for Alzheimer’s disease[J]. Alzheimers Dement.

[21] Petersen RC, Roberts RO, Knopman DS (2009). Mild cognitive impairment: ten years later[J]. Arch Neurol.

[22] Molinuevo JL (2014). Measuring decline in prodromal AD: a pike to hike[J]. J Neurol Neurosurg Psychiatry.

[23] Zhou Y, Qun-Xu Qin L D (2011). A primary study of diffusion tensor imaging-based histogram analysis in vascular cognitive impairment with no dementia[J]. Clin Neurol Neurosurg.

[24] Taler V, Monetta L, Sheppard C (2019). Semantic function in mild cognitive impairment[J]. Front Psychol.

[25] Hampel H, Mesulam MM, Cuello AC (2018). The cholinergic system in the pathophysiology and treatment of Alzheimer’s disease[J]. Brain.

[26] Nemy M, Cedres N, Grothe MJ (2020). Cholinergic white matter pathways make a stronger contribution to attention and memory in normal aging than cerebrovascular health and nucleus basalis of Meynert[J]. NeuroImage..

[27] Salvadori E, Poggesi A, Donnini I (2020). Association of nimodipine and choline alphoscerate in the treatment of cognitive impairment in patients with cerebral small vessel disease: study protocol for a randomized placebo-controlled trial—the CONIVaD trial[J]. Aging Clin Exp Res.

[28] Wang SN, Wang JF, Shi QL (2020). Analysis the relationship of cholinergic pathway damage and cerebral cortex structure change in patients with cognitive impairment accompanied by white matter hypertensity[J]. Chinese Journal of Contemporary Neurology and Neurosurgery.

[29] Mcaleese KE, Alafuzoff I, Charidimou A (2016). Post-mortem assessment in vascular dementia: advances and aspirations[J]. BMC Med.

